# Erratum to: Long non-coding RNA ANRIL is upregulated in hepatocellular carcinoma and regulates cell proliferation by epigenetic silencing of KLF2

**DOI:** 10.1186/s13045-017-0513-0

**Published:** 2017-07-27

**Authors:** Ming-de Huang, Wen-ming Chen, Fu-zhen Qi, Rui Xia, Ming Sun, Tong-peng Xu, Li Yin, Er-bao Zhang, Wei De, Yong-qian Shu

**Affiliations:** 1 Department of Medical Oncology, Huai’an First People’s Hospital, Nanjing Medical University, Huai’an City, Jiangsu Province, 223301 People’s Republic of China; 2 Department of Oncology, Jining No.1 People’s Hospital, No.6, Jiankang Road, Jining City, Shandong Province 272011 People’s Republic of China; 3 Department of Hepatopancreatobiliary Surgery, Huai’an First People’s Hospital, Nanjing Medical University, Huai’an City, Jiangsu Province, 223300 People’s Republic of China; 4 Department of Biochemistry and Molecular Biology, Nanjing Medical University, Nanjing City, Jiangsu Province People’s Republic of China; 5 Department of Oncology, First Affiliated Hospital, Nanjing Medical University, Nanjing City, Jiangsu Province People’s Republic of China

## Erratum

This article [1] was unintentionally published twice in this journal, by the same authors.

The following [1] should be considered the version of record and used for citation purposes: “Huang M-d, Chen W-m, Qi F-z, Xia R, Sun M, Xu T-p, Yin L, Zhang E-b, De W, Shu Y-q, Long non-coding RNA ANRIL is upregulated in hepatocellular carcinoma and regulates cell proliferation by epigenetic silencing of KLF2. Journal of Hematology & Oncology 2015, 8:57 DOI: 10.1186/s13045-015-0153-1”.

The duplicate [2]: “Huang M-d, Chen W-m, Qi F-z, Xia R, Sun M, Xu T-p, Yin L, Zhang E-b, De W, Shu Y-q Long non-coding RNA ANRIL is upregulated in hepatocellular carcinoma and regulates cell apoptosis by epigenetic silencing of KLF2. Journal of Hematology & Oncology 2015, 8:50 DOI: 10.1186/s13045-015-0146-0” is to be ignored.

BioMed Central apologizes to the readers of the journal for not detecting the duplication during the publication process.
